# A rapid and simple method for the extraction of biogenic silica (BSi) in phytolith-poor sediments and soils

**DOI:** 10.1016/j.mex.2024.102634

**Published:** 2024-02-24

**Authors:** Arnaud Mazuy, Vincenza Ferrara, Anneli Ekblom, Claire Delhon

**Affiliations:** a Université Côte d'Azur, CNRS, CEPAM, France; b Department of Archaeology and Ancient History, Uppsala University - Engelska Parken, Thunbergsvägen 3H, Uppsala 751 26, Sweden; c Department of Human Geography, Stockholm University - Geovetenskapens hus, Svante Arrhenius väg 8, Frescati, Stockholm 106 91, Sweden

**Keywords:** Phytoliths, Historical ecology, Agrosystems, Time-efficient, easy and safe extraction of biogenic silica (BSi) from phytolith-poor sediments

## Abstract

Phytoliths can be used to reconstruct human-nature dynamics over the long term (from decennial to centennial and millennial time scales) and may capture activities that cannot be reconstructed through other proxies. Phytoliths consist of fossil biogenic silica (BSi), formed in plant organs and then released into the soil with plant decay. When working in environmental contexts where the phytolith signal is highly diluted, as is the case in environments with a long history of land use, animal-plant interactions and open woody environments, the extraction of phytoliths remains a challenge. To address this issue, we developed an efficient method for the extraction of biogenic silica (BSi) from sediments and soils of contexts characterised by the long-term human and animal presence and disturbance, such as remnants of old agroforestry systems.

The method we developed has a number of advantages, including:

• An easy and time-efficient methodology to perform (with an overall processing time of 1.5/2 days for a batch of 16 samples)
• An extraction method free from dangerous chemicals
• A method amenable to non-experts without a prior background in lab extraction procedures.

## Graphical abstract

**Figure:**
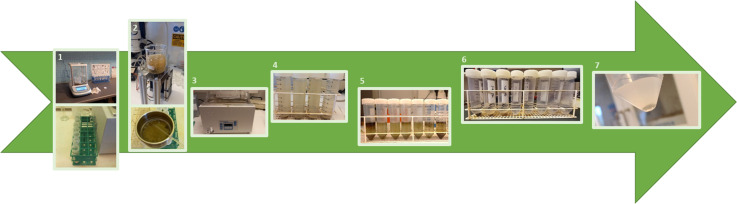

Specifications table

**Table:** 

Subject area:	Environmental Science
More specific subject area:	*Biogenic silica (BSi) extraction from soil and sediments; phytoliths extraction*
Name of your method:	*Time-efficient, easy and safe extraction of biogenic silica (BSi) from phytolith-poor sediments*
Name and reference of original method:	Kelly, E. F. (1990), Methods for extracting opal phytoliths from soil and plant material, report, Dep. of Agron., Colorado State Univ., Fort Collins. Piperno, D. (2006), Phytoliths: A Comprehensive Guide for Archaeologists and Paleoecologists, Altamira Press, Lanham.
Resource availability:	*N/A*

## Method details

### Introduction

Silicate minerals are abundant in the Earth's crust and, due to weathering under proper physical and chemical conditions, they dissolve into the soil as orthosilicic acid (DSi). DSi is taken up by plants and, depending on the plant type, it is deposited as biogenic silica (BSi) rich bodies called phytoliths, in the cell, intercellular walls and in various plant organs. After the degradation of the plant, the BSi accumulated as phytoliths return to the soil [4].

Even though phytoliths cannot provide detailed taxonomic information for all the plant families, they are preserved in environmental conditions where other organic residues decay [38], their signal can thus represent plants that are not represented by the pollen or seeds records [42]. Furthermore, being subject to *in situ* deposition, the signal given by these indicators is local [13].

Fossil phytoliths assemblages from soils have been used broadly in paleoecology for the reconstruction of past vegetation patterns and dynamics [2,16,39,53], habitat characterisation [53], plant-animal coevolution [45], erosion patterns [24], paleoclimatic studies [9,21,29,32,[45], [57]], evolutionary history of grasses [45].

In addition, since phytoliths produced from grasses allow the identification at the subfamily and sometimes genus level [10], they provide important ecological information on past land uses, agricultural practices and management regimes [[3], [12], [15],^18^,^20^,^39^].

Last but not least, studies on phytoliths have been applied in research on sustainable agriculture [19,27,33,43]. Land use and its changes impact significantly the Si cycle in ecosystems [11,35,50,51]. Since plants and soils act as Si “filter” ([55], p. 243), transforming the DSi originated from mineral weathering into biogenic Si (BSi) and then returning it to the soil when plants decay, changes and disruptions caused in vegetation and soils by land use activities (such as export of biomass due to harvest) impact the terrestrial Si cycle (in both quantity and distribution, cf. [4]).

Compared to their concentration in certain soils from very specific environments (e.g. grassy environment, archaeological sites) rich in phytoliths, the concentration of phytoliths in environments with a long history of land use, animal-plant interactions and open woody environments (such as orchards and scrublands) can be relatively low [7]. This is due to the fact that, in addition to the biogenic silica (BSi) strongly decrease along the land use change gradient (from forest to croplands) [54], some environments or farming systems rely on vegetation that produces low phytolith amounts (e.g. arboriculture) and thus result in soils with low phytolith content.

In contexts where the phytolith signal is highly diluted, such as the ones mentioned above, the extraction of phytoliths remains a challenge. This is particularly the case for many temperate environmental contexts based on remnants of old agroforestry systems, which are scientifically relevant to investigate the impacts of long term land use dynamics on the current state of those agroecosystems.

To address this issue and ensure the recovery of the phytoliths preserved in the above-described environmental contexts, we developed a time-efficient, easy and safe method for the extraction of biogenic silica (BSi) from soils derived from open agricultural and environmental contexts, characterised by a long-term history of human and animal presence and a low phytolith accumulation rate.

With these considerations, our method opens up the possibility to push further research on the use of biogenic Si as a proxy to understand the impacts of past land use practices on current soils and ecosystem conditions.

## Method details

Phytoliths extraction was conducted in the Fluorväte Lab of the Department of Earth Sciences at Uppsala University (Sweden).

Soil samples from five vertical profiles of 1.5 m depth following a slope transect were collected at different layers (distance between each layer = 10 cm), for a total of seventy-five samples. The study site, on the island of Sicily (Italy), is characterised by the presence of olive trees (*Olea europaea* L.) which, based on their size and features, are several centuries old. Phytolith extraction was conducted as integration to historical ecology investigations done in the study site (cf. [17]), with the intent, first of all, to assess the state of preservation of biogenic silica in this type of environmental context and, secondly, to evaluate the possibility to develop integrative methods that may allow a better understanding of the different land uses practices in the area over time (cf. [5,31] on the application of phytoliths in historical ecology). The profiles studied may cover a period prior to olive cultivation and shed light on the impact of olive cultivation and its changes along centuries (from a system based mainly on agroforestry to olive monoculture heavily relying on mechanisation) on the current state of this ecosystem.

In our method, the extraction of phytoliths from sediments and soils aims to isolate the maximum quantity possible of phytoliths from the organic and mineral components of the sample. In order to extract phytoliths, in our method the samples are first deflocculated through magnetic stirring, then the fine fraction (200 µm) of the sediment is treated respectively with concentrated hydrochloric acid (to remove carbonates) and then with potassium hydroxide (to remove organic matter) in a hot bath. Phytoliths are then extracted by heavy liquid flotation (using sodium polytungstate, SPT) and treated with hydrogen peroxide in a hot bath to further remove residues of microcharcoals and organic matter.

## Materials and reagents

• 50 ml centrifuge tubes with cap (VWR)
• 250 ml glass beakers (Pyrex® n. 1003)
• 200 µm sieves (VWR)
• Glass Pasteur pipettes (150 ml) (VWR)
• Nitrile gloves (VWR)
• Vortex mixer (IKA Janke & Kunkel Labortechnik VF2)
• Magnetic stirrer (self-fabrication, prototype)
• Benchtop centrifuge (Jouan B4i)
• Water Bath (VWR, VWB2 5)
• Fresh demineralised water
• Hydrochloric acid puriss. p.a., ACS reagent, reag. ISO, reag. Ph. Eur. (Honeywell)
• Potassium hydroxide, pellets, extra pure, Ph. Eur., BP, NF (SCHARLAU)
• Sodium metatungstate hydrate, crystalline (Thermo Fischer Scientific)
• Hydrogen peroxide 30% w/v (100 volumes), extra pure (Thermo Fisher Scientific)
• Glycerol AB Unimedic

Note: This list does not include any small generic laboratory equipment that is assumed to be available. Chemicals and other components are standard and can be ordered from any reliable company.

### Procedure

**Step 1**. Weigh untreated samples to 3.5 g into 50 ml centrifuge tubes, add fresh demineralised water and swirl with vortex for 10 s.

3.5 g is a suitable average quantity (cf. quantities used, e.g., in [[14], [40],^42^,^53^]) for the type of environmental context we work with.

**Step 2**. Deflocculate for 5/6 h in fresh demineralised water (250 ml glass beakers) with magnetic stirring**,** then sieve at 200 µm in 50 ml centrifuge tubes, centrifuge 5 min at 2000 rpm. Discard the supernatant.

Fresh demineralised water has a high capacity of deflocculating when coupled with magnetic stirring. Furthermore, such a procedure can guarantee a faster and more controlled approach to deflocculation than through the use of a chemical deflocculant.

**Step 3**. Add Hydrochloric acid (HCl) (33%), drop by drop, until 14 ml and place the tubes in the water bath at 80 °C for 15 min. Centrifuge 5 min at 2000 rpm. Discard the supernatant.

Rinse 3 times the samples with fresh demineralised water: add fresh demineralised water, swirl with vortex for 5 s or until the sample is perfectly diluted, centrifuge 5 min at 2000 rpm, discard the supernatant. Repeat the process 3 times.

The aim of Step 3 is to remove calcium carbonates and break up particles bound with carbonates.

**Step 4**. Add 7 ml of Potassium hydroxide (KOH) (10%), swirl with vortex for 5 s (or until the sample is perfectly diluted) and place the tubes in the water bath at 80 °C for 15 min. Centrifuge 5 min at 2000 rpm. Discard the supernatant.

Rinse 6 or more times with fresh demineralised water (swirl with vortex for 5 s or until the sample is perfectly diluted, then centrifuge for 5 min at 2000 rpm) until the supernatant is clear, then discard the supernatant.

**(Optional)** Pipette out and discard the remaining drops of supernatant.

This step aims to remove soil organic matter (SOM) content present in the samples. There is no risk that KOH dissolves phytoliths, if the step will be performed at the specified T°, duration, concentration and with a rapid rinsing ensured (also noted in [41,42]).

**Step 5.** Gravimetric separation of phytoliths with Sodium metatungstate hydrate Na_6_ (Density = 2.35).

Add a solution of Na_6_ (*D* = 2.35) directly to the samples using the following amount: Volume (Na_6_) = 2 x Volume of the sediment in the samples.

Swirl the samples with vortex for at least 15 min and centrifuge for 5 min at 2000 rpm. Recover the supernatant (which contains the phytoliths) in a new tube. Save the leftover for further separation (if necessary) and store them at refrigerated temperature (4 °C).

There is no need to dry the samples before the gravimetric separation. The potential presence of supernatant from the previous step (demineralised water, Step 4) will not affect the gravimetric separation process when the density of the Na_6_ solution has been correctly calibrated with a precise densimeter and an adequate homogenisation in the samples reached (through at least 15 min of swirling).

Dilute the density of the supernatant (which contains the phytoliths) with fresh demineralised water, using the following amount: Volume (water) = 2 x Volume (Na_6_ supernatant) (e.g. for 4 ml of Na_6_ supernatant, we add 8 ml of fresh demineralised water). Swirl the samples with vortex for at least 5 min and centrifuge for 5 min at 3000 rpm. Repeat this diluting process twice.

Rinse the samples 3 times with fresh demineralised water (swirl with vortex for 5 s, then centrifuge for 5 min at 2000 rpm). Sodium metatungstate hydrate Na6 is a non-toxic and low-viscosity heavy liquid, which is very soluble in fresh demineralised water. For such reason, it is easily washable with only fresh demineralized water.

Recycle diluted Na_6_ solution from the rinsing process.

**Step 6**. Add 5 ml of Hydrogen peroxide H₂O₂ (30%) to the samples, swirl with vortex for 10 s and place the tubes in the water bath at 80 °C for 1 hour (if H₂O₂ gives a strong reaction, reduce the time of the bath). Centrifuge 5 min at 2000 rpm. Discard the supernatant.

Repeat the process 3 times, if needed. Step 6 is meant to remove potential leftovers of microcharcoals (if present in the samples) and soil organic matter (SOM).

Rinse the sample 2/3 times with fresh demineralised water (swirl with vortex for 5 s, then centrifuge for 5 min at 2000 rpm).

**Step 7.** Dry the phytoliths at room temperature (or in a drying oven at moderate temperature) and save in Glycerol at room temperature.

The specific step of phytoliths extraction (step 5 in our procedure) is generally done through gravimetric separation [23,42] since their specific gravity range (1.5 – 2.3) allows them to flotate compared to other minerals when immersed in heavy liquid. As reviewed by Strömberg et al. [52], Rashid et al. [46], Cabanes [6] and Shoda et al. [49], there are several available methods to extract phytoliths from soils and sediments [1,8,22,[25], [28],^30^,^36^,^38^,^44^,^48^,^56^], which vary greatly in the initial amount of sample needed (from tens of grams to tens of milligrams), and the duration of the process (from several days to few minutes). However, all of them have two basic steps in common:

(1) Pre-treatment of the sample for the disaggregation of the sediments, and removal of carbonates, organic matter and clay;
(2) Separation of biogenic silica from the mineral fraction through heavy liquid gravity separation.

While our protocol follows the principle of gravimetric separation [23], it differs from standard wet-ashing procedures [42] in the following steps:

- The initial disaggregation of clay in the sediment is done via magnetic stirring with fresh demineralised water and not using a deflocculant solution, acting as a dispersing agent overnight [42]. This guarantees the same results as chemical deflocculation, at the same time faster and more controlled.
- Clay removal is done along the process simultaneously with steps removing carbonates and organic matters, so to eliminate a step just dedicated to clay removal (as done instead, for instance, in Piperno [42] through gravity sedimentation or in Pearsall [37] and Lentfer et al. [26] through centrifuge sedimentation).
- Another difference between our method, compared to Piperno [42], is in the use of H₂O₂ and KOH to remove organic matter, as well as in the order these procedures are performed.

While in Piperno [42] organic matter is removed with HNO₃ + KC1O3 or H₂O₂ 30% (overnight), we perform this step with KOH 10% (allowing to remove clay as well), Instead, we use H₂O₂ 30% (in just one hour of water bath and not overnight) only on the extracted fraction from the gravity separation, with the aim to further remove potential leftovers of microcharcoals and soil organic matter (SOM).

Such a procedure allows us to have faster and clean results, while at the same time reducing significantly the amount of polluting and hazardous chemicals used. Other protocols (e.g. Rosen, [47]) suggest a quick and efficient removal of organic by burning the sediments in a muffle furnace at 500° degrees. In our approach, we avoid burning procedures since we do not want to transfer the samples from the tubes, thus avoiding risks of sample dispersal and contamination (also noted by [6]).

## Method validation

The efficiency of our extraction method was tested by assessing the number of phytoliths extracted.

**Fig. 1:**
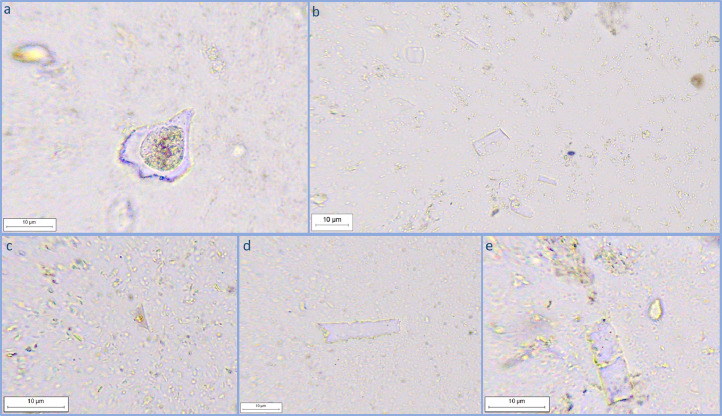
Images of common phytolith morphotypes extracted from the processed samples: bulliform (a); elongated, rondel and crenate (b); rondel (c); elongated (d); elongated (e).

The extracted phytoliths were mounted on microscope slides using glycerol and analysed at the UMR 7264 CEPAM CNRS-Université Côte d'Azur (Nice, France). The slides were observed under a Leica DMRB™ microscope from 400× to 1000× magnification (Leica Microsystems, Germany). We recommend the use of cross-polarised light microscopy for the positive identification of silica bodies. For each sample, we endeavoured to count at least 200 phytoliths and classify them following the International Code for Phytolith Nomenclature 2.0 [34]. The samples extracted with the method presented here gave excellent results in terms of phytoliths abundance and in terms of the quality of the extraction process itself: preservation condition of the phytoliths was good and no damage could be observed on the silica bodies from the extraction process (Fig. 1). The final extraction of phytoliths was also very clean from other particles. The extraction method developed and used is therefore efficient for the extraction of biogenic silica (BSi) in soils from open environmental and agricultural contexts where phytoliths are few. The presented extraction method is relatively simple and rapid in allowing an optimised phytoliths extraction and, therefore, it is suitable also for archaeological sites and palaeoecological contexts with low phytolith concentration.

## Ethics statements

No ethical considerations were required.

## CRediT authorship contribution statement

**Arnaud Mazuy:** Conceptualization, Methodology, Writing – review & editing. **Vincenza Ferrara:** Conceptualization, Investigation, Data curation, Validation, Writing – original draft. **Anneli Ekblom:** Conceptualization, Supervision, Resources, Funding acquisition. **Claire Delhon:** Conceptualization, Methodology, Validation, Supervision.

## Declaration of competing interest

The authors declare that they have no known competing financial interests or personal relationships that could have appeared to influence the work reported in this paper.
